# Comparing the Infection Biology of *Plasmodiophora brassicae* in Clubroot Susceptible and Resistant Hosts and Non-hosts

**DOI:** 10.3389/fmicb.2020.507036

**Published:** 2020-10-16

**Authors:** Lijiang Liu, Li Qin, Xiaohui Cheng, Yi Zhang, Li Xu, Fan Liu, Chaobo Tong, Junyan Huang, Shengyi Liu, Yangdou Wei

**Affiliations:** ^1^Key Laboratory of Biology and Genetic Improvement of Oil Crops, Ministry of Agriculture and Rural Affairs, Oil Crops Research Institute, Chinese Academy of Agricultural Sciences, Wuhan, China; ^2^Department of Biology, University of Saskatchewan, Saskatoon, SK, Canada

**Keywords:** clubroot disease, *P. brassicae*, infection biology, non-host, resistant host

## Abstract

The potential infection biology of *Plasmodiophora brassicae* in resistant hosts and non-hosts is still not completely understood. Clubroot resistance assay on European clubroot differentials (ECD) set revealed that ECD10 (*Brassica napus*) and ECD4 (*Brassica rapa*) show a complete resistance to the tested *P. brassicae* isolate in contrast to highly susceptible hosts Westar (*B. napus*) and ECD5 (*B. rapa*). Previously, we used fluorescent probe-based confocal microscopy (FCM) to refine the life cycle of *P. brassicae* and indicate the important time points during its infection in *Arabidopsis*. Here, we used FCM to systematically investigate the infection of *P. brassicae* in two resistant host species ECD10 and ECD4 and two non-host crops wheat and barley at each indicated time points, compared with two susceptible hosts Westar and ECD5. We found that *P. brassicae* can initiate the primary infection phase and produce uninucleate primary plasmodia in both resistant hosts and non-hosts just like susceptible hosts at 2 days post-inoculation (dpi). Importantly, *P. brassicae* can develop into zoosporangia and secondary zoospores and release the secondary zoospores from the zoosporangia in resistant hosts at 7 dpi, comparable to susceptible hosts. However, during the secondary infection phase, no secondary plasmodium was detected in the cortical cells of both resistant hosts in contrast to massive secondary plasmodia present in the cortex tissue of two susceptible hosts leading to root swelling at 15 dpi. In both non-host crops, only uninucleate primary plasmodia were observed throughout roots at 7 and 15 dpi. Quantitative PCR based on DNA revealed that the biomass of *P. brassicae* has no significant increase from 2 dpi in non-host plants and from 7 dpi in resistant host plants, compared to the huge biomass increase in susceptible host plants from 2 to 25 dpi. Our study reveals that the primary infection phase in the root epidermis and the secondary infection phase in the cortex tissue are, respectively, blocked in non-hosts and resistant hosts, contributing to understanding of cellular and molecular mechanisms underlying clubroot non-host and host resistance.

## Introduction

Clubroot disease, caused by the soil-borne protist pathogen *Plasmodiophora brassicae*, spreads over 60 countries and results in huge yield and economic losses worldwide (Dixon, 2014). Furthermore, the resting spores of *P. brassicae* can survive in soil as long as 20 years (Wallenhammar, 1996), making the *P. brassicae*-infested soil unsuitable for the cultivation of cruciferous crops. Presently, how to efficiently and durably control the clubroot disease still poses a great challenge.

One important feature of *P. brassicae* is the pathogenic specialization which could be determined by various sets of clubroot differential hosts (Ayers, 1957; Williams, 1966; Buczacki et al., 1975; Somé et al., 1996; Strelkov et al., 2018). The Williams clubroot differential set (WCD) and European clubroot differential set (ECD) have been widely employed to determine the pathotypes of *P. brassicae* worldwide, and accordingly, 7 pathotypes in China, 6 pathotypes in Canada, 8 pathotypes in France, and 23 pathotypes in Australia have been identified (Manzanares-Dauleux et al., 2001; Donald et al., 2006; Chai et al., 2014; Strelkov et al., 2018). It greatly contributes to the understanding of the global population diversity and heterogeneity of *P. brassicae*. On the other hand, WCD or ECD *per se* provides important resistant resources for *Brassica* crop resistance breeding and probing into resistance mechanism.

The soil-infested nature of *P. brassicae* makes it impracticable to control the clubroot disease by using chemical treatments on a large scale. Therefore, it is key to exploit and utilize natural clubroot-resistant resources. Clubroot-resistant materials are widely present in *Brassicaceae* species revealed by a large screening of germplasm (Hirai, 2006; Diederichsen et al., 2009; Piao et al., 2009; Jakir Hasan et al., 2012; Peng et al., 2014; Farid et al., 2020). Presently, more than 20 major clubroot disease-related loci have been identified in *Brassica* vegetables, and several resistant genes have been isolated by map-based cloning (Mehraj et al., 2020). In general, clubroot-resistant loci and genes from *B. rapa* (A genome) are pathotype-specific and monogene-controlled, while those from *Brassica oleracea* (C genome) are always pathotype-non-specific and controlled by polygene. Among isolated resistant genes, two genes *Crr1a* and *CRa* from *B. rapa* were functionally validated and found to be nucleotide binding site-leucine rich repeat (NBS-LRR) resistance genes. Considering the intracellular parasitic feature of *P. brassicae*, the underlying mechanisms of clubroot-resistant genes may be different and remain to be uncovered in the future.

The hosts of clubroot disease always limit in the members of family *Brassicaceae* (Dixon, 2004), despite occasional cases of which *P. brassicae* was reported to successfully infect some non-cruciferous species (Ludwig-Müller et al., 1999). To efficiently and durably control the clubroot disease, attempts of using clubroot non-hosts as baits to reduce the soil-infested resting spores have been made in several cases (Friberg et al., 2006; Ahmed et al., 2011; Hwang et al., 2015). However, potential infection, growth, and reproduction of *P. brassicae* within root tissues of non-hosts have not been understood, largely due to the lack of sufficient techniques to visualize or detect *P. brassicae* infection structures within plant tissues.

The life cycle of *P. brassicae* is generally divided into the primary infection in root epidermis and the secondary infection in cortex tissue, of which, the later infection phase is responsible for clubroot disease in susceptible hosts (Tommerup and Ingram, 1971; Ingram and Tommerup, 1972; Kageyama and Asano, 2009; Liu et al., 2020). Previously, we have successfully utilized fluorescent probe-based confocal microscopy (FCM) and transmission electron microscopy to clarify the life cycle of *P. brassicae* (Liu et al., 2020). In this study, we mainly used FCM to investigate the infection biology of *P. brassicae* in two resistant host species *B. rapa* ECD4 and *B. napus* ECD10 and two non-host crops wheat and barley, compared with susceptible host species *B. rapa* ECD5 and *B. napus* Westar. We have quantified the biomass of *P. brassicae* during its infection with quantitative PCR (qPCR) based on DNA and finally determined the specific life stages of *P. brassicae* that were blocked in non-hosts and resistant hosts. Exploring the clubroot non-hosts as an integrative management to control clubroot disease is also discussed.

## Materials and Methods

### Plant Growth and Pathogen Inoculum

The seeds of European clubroot differentials (ECD) set were from the courtesy of Dr. G.R. Dixon, Dr. Sarah Trinder and their colleagues (Warwick Genetic Resources Unit, The University of Warwick, Warwick, United Kingdom). The seeds of *B. napus* accession Westar, wheat accession Conway, and barley accession Silky were maintained in our lab. All plants were grown under controlled conditions with 21–23°C and 16 h light and 8 h darkness. The isolate of *P. brassicae* used in this study is from the diseased canola plants in the field (Edmonton, Alberta, Canada). Maintenance of *P. brassicae*, resting spore preparation, and inoculation were performed as previously described (Liu et al., 2020). Ten-day-old seedlings were inoculated with 1 ml resting spore suspension (1.0 × 10^8^ spores/ml) of the *P. brassicae* isolate.

### Identification of Clubroot-Resistant Germplasm From ECD Set

The clubroot resistance of all 15 ECD accessions was tested with the *P. brassicae* isolate used in this study. Ten-day-old seedlings of each ECD accession were inoculated with resting spores of *P. brassicae* as descriptions mentioned above. At 25 dpi, all plant roots were collected for the clubroot severity investigation according to a standard disease scale 0 to 3: 0, no clubs; 1, a few small clubs on the lateral or main root; 2, moderate clubs on the lateral or main root; 3, severe clubs on the main root. The disease index (DI) of each ECD accession was calculated based on the mean value of all tested seedlings. DI = 0, immune; 0 < DI ≤ 10, highly resistant; 10 < DI ≤ 30, resistant; 30 < DI ≤ 50, susceptible; DI > 50, highly susceptible. The *P. brassicae* isolate used in this study was coded according to the binary nomenclature of ECD.

### Confocal Laser Scanning Microscopy

Three individual inoculated plant roots of two resistant hosts ECD10 and ECD4, two non-host crops wheat and barley, and two susceptible hosts Westar and ECD5 were, respectively, collected at 2, 7, and 15 dpi and subject to fluorescent probe-based confocal microscopy (FCM). For observation of *P. brassicae* parasites during the primary infection, the lower part of main root and three lateral roots of each plant harvested at 2 and 7 dpi were subsequently collected and mounted on the slides. The solution mixed with several live cell dyes was dropped on the roots and the cover slide was placed on top. After staining for 10 min, the sample was observed under confocal microscope, and the root elongation zone was mainly focused. To observe *P. brassicae* parasites in the cortical cells during the secondary infection, the swollen roots of susceptible hosts and the main root and all main lateral roots of resistant hosts collected at 15 dpi were sectioned by hand. The resultant sections were mounted on the slides and stained with solutions of mixed live cell dyes for 10 min. Then, the sections were examined under confocal microscope. Live cell dyes HCS LipidTOX^TM^ Green Neutral Lipid Stain (HLG, Thermo Fisher Scientific Canada), 4,6-Diamidine-2-phenylindole dihydrochloride (DAPI, Sigma-Aldrich, Canada), and FM4-64 (Thermo Fisher Scientific, Canada) were used to, respectively, label the lipid droplets, nuclei, and membrane structures of *P. brassicae* as previously described (Liu et al., 2020). The excitation wavelength and emission filter set for HLG, DAPI, and FM4-64 were performed following previous methods (Liu et al., 2020). Images were harvested with a Zeiss LSM Meta 510 (Carl Zeiss, Germany). The contrast and brightness levels were optimized for visualizing *P. brassicae* structures in susceptible host species and the same setting was performed for the visualization of *P. brassicae* in resistant hosts and non-hosts by the “reuse” function implanted in Zeiss LSM Meta 510.

### Quantitative Determination of *P. brassicae* Biomass

Ten-day-old seedlings of resistant hosts ECD10 and ECD4, non-host wheat and susceptible hosts Westar and ECD5 were inoculated with 1 ml 1 × 10^8^/ml resting spores of *P. brassicae*. After inoculation, the roots of each accession were collected at 0, 2, 7, 15, and 25 dpi with three repeats. DNA extraction was performed with a plant DNA extraction kit (Qiagen, Canada) following the user’s manual. DNA concentration and purity were monitored on 1% agarose gel. For quantitative determination of *P. brassicae*, qPCR analysis was performed using the primer pair designed by the ITS1 and 5.8S rDNA sequence of *P. brassicae* (Supplementary Table S2), as previously described (Cao et al., 2007). The DNA samples were diluted to 20 ng/μl with sterile distilled water and 10 μl reaction with three repeats for each sample were analyzed with CFX96 Real-Time PCR Detection System (Bio-Rad, United States). Each reaction was mixed with 5 μl of 2 × SYBR Green Super mix (Bio-Rad, United States), 1 μl of sample DNA, 0.15 μl of forward primer, 0.15 μL of reverse primer (10 μmol/l), and 3.7 μl sterile distilled water. The program was performed as follows: denaturation at 95°C for 4.5 min followed by 40 amplification cycles of 95°C for 15 s, 56°C for 15 s, and 72°C for 15 s. The actin gene of *B. napus*, *B. rapa*, and wheat was, respectively, used as the internal control for normalization, and the primers were listed in Supplementary Table S2. Data for quantification analyses are presented as mean ± standard error (SE). The statistical analyses were performed by one-way analysis of variance (ANOVA) test (^∗^*p* < 0.05).

## Results

### Identifying Clubroot-Resistant Germplasm From European Clubroot Differential Set

**FIGURE 1:**
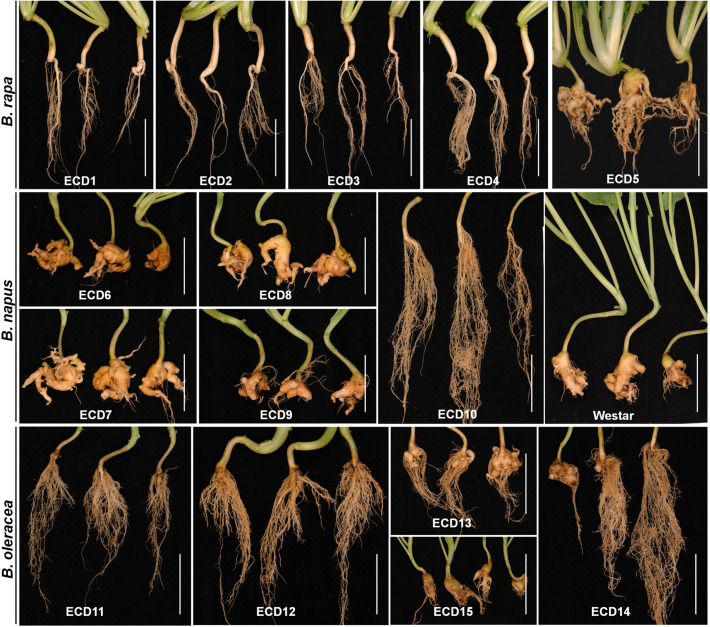
Clubroot resistance assay on ECD accessions. Ten-day-old seedlings of each ECD accession were inoculated with resting spores of *P. brassicae*, and all plant roots were collected at 25 dpi and assayed for the clubroot severity. *B. napus* var. Westar was used as the susceptible control. Scale bars: 5 cm.

Plants of all ECD accessions were inoculated with resting spores of *P. brassicae* and evaluated for clubroot resistance at 25 dpi when plant roots of susceptible accessions *B. rapa* ECD5 and *B. napus* Westar showed severe clubroot symptoms (Figure 1). For *B. rapa* accessions, all roots of ECD1 or ECD4 were club-free and free of gall showed a complete resistance against the *P. brassicae* isolate used in this study (Figure 1 and Supplementary Table S1). Only one root of ECD2 or ECD3 was observed with a few small clubs and determined with the disease index (DI) of 6.7 and 2.8, respectively. In the case of *B. napus* accessions, all roots of ECD6, ECD7, ECD8, and ECD9 showed severe clubroot disease and determined with a DI ranging from 83.3 to 100 (Figure 1 and Supplementary Table S1). In contrast, most roots of ECD10 were club-free, while three roots were severely diseased, suggesting a potential genetic heterogeneity (Supplementary Table S1). In total, the clubroot severity of ECD10 was determined with 23.1, showing a highly clubroot resistance. As for *B. oleracea* accessions, the clubroot severity is variable and ranged from a disease scale 0 to 3 (Figure 1 and Supplementary Table S1), similar to previous studies of which the clubroot resistance underlying *B. oleracea* species is always partial and depends on the cumulative actions of many genes and the environment (Hirai, 2006; Diederichsen et al., 2009; Piao et al., 2009). Based on the ECD nomenclature, this inoculated isolate of *P. brassicae* is coded ECD16/15/28 (Supplementary Table S1).

### *P. brassicae* completes the primary infection phase in clubroot-resistant species *B. napus* and *B. rapa*

**FIGURE 2:**
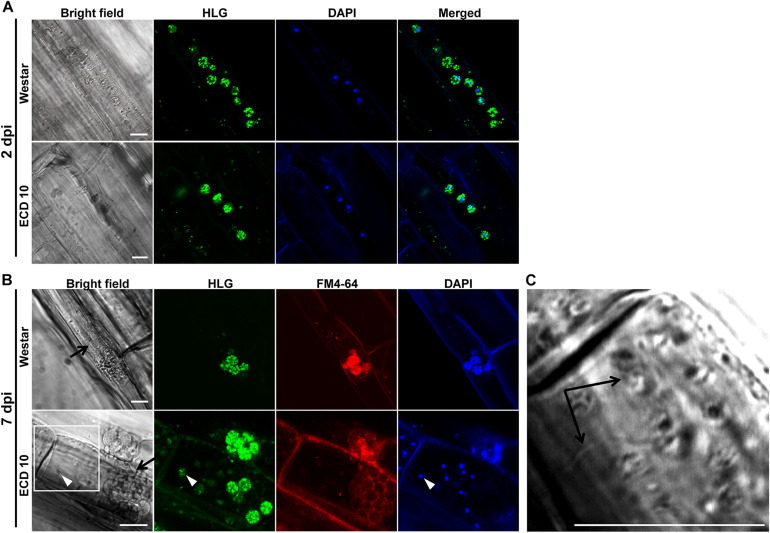
The primary infection of *P. brassicae* on susceptible and resistant host plants of *B. napus.* The primary infection of *P. brassicae* was investigated in susceptible host plants of Westar and resistant host plants of ECD10. **(A)** FCM images of the uninucleate primary plasmodia in the root epidermal cells at 2 dpi. Live cell dyes HLG and DAPI were used to label lipid droplets and nuclei of *P. brassicae* parasites, respectively. **(B)** FCM images of zoosporangia and secondary zoospores at 7 dpi. In addition to HLG and DAPI, live cell dye FM4-64 was used to label the plasma membrane of *P. brassicae* parasites. Empty zoosporangium was indicated with a black arrow in the bright field. A free secondary zoospore was pointed with a white arrowhead in the lower panel. The part highlighted with a white box in the bright field (lower panel) was further enlarged for a view in **C**. **(C)** A free secondary zoospore was profiled. The head and long flagellum of secondary zoospore were indicated with black arrows. Scale bars: 10 μm.

**FIGURE 3:**
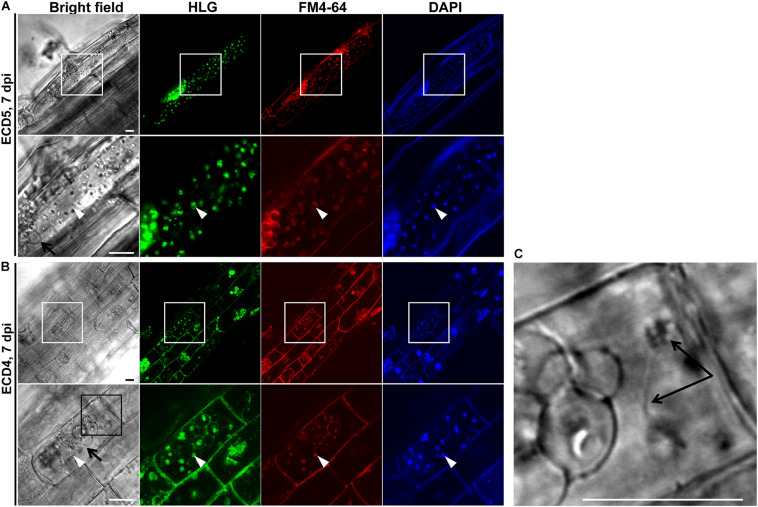
The primary infection of *P. brassicae* on susceptible and resistant host plants of *B. rapa.* The primary infection of *P. brassicae* was investigated in susceptible host plants of ECD5 and resistant host plants of ECD4. **(A)** FCM images of the zoosporangia and secondary zoospores in the root epidermal cells of susceptible host plants at 7 dpi. Live cell dyes HLG, DAPI and FM4-64 were used to label lipid droplets, nuclei, and plasma membrane of *P. brassicae* parasites, respectively. The part highlighted with a white box was enlarged for a view in the lower panel. **(B)** FCM images of the zoosporangia and secondary zoospores in the root epidermal cells of resistant host plants at 7 dpi. Live cell dyes HLG, DAPI, and FM4-64 were used to label lipid droplets, nuclei, and plasma membrane of *P. brassicae* parasites, respectively. The part highlighted with a white box was enlarged for a view in the lower panel. The part highlighted with a black box in the bright field (lower panel) was further enlarged for a view in **C**. **(C)** A free secondary zoospore was profiled. The head and long flagellum of secondary zoospore were indicated with black arrows. Scale bars: 10 μm.

Based on above results, two clubroot-resistant host species *B. napus* ECD10 and *B. rapa* ECD4 were used to investigate the potential infection of *P. brassicae* compared with susceptible hosts *B. napus* Westar and *B. rapa* ECD5. Given the fact that the infection of *P. brassicae* is always asynchronous and very limited root hairs or epidermal cells were found to be successfully infected by *P. brassicae* even on the susceptible hosts during the primary infection (Liu et al., 2020), we mainly focused on the epidermal cells of the root elongation zone where it is easier to observe the colonized *P. brassicae*. The live cell dyes HLG, DAPI, and FM4-64 have been successfully used to, respectively, label the lipid droplets, nuclei, and membrane structures of *P. brassicae* and visualize the pathogen under confocal microscope examination (Liu et al., 2020). We firstly used the live cells HLG and DAPI to label and visualize *P. brassicae* at 2 dpi and found that several lipid droplet-enriched uninucleate primary plasmodia were always detected in an single root epidermal cell of resistant host *B. napus* ECD10, comparable to the susceptible host *B. napus* Westar (Figure 2A), indicating a successful initiation of the primary infection in resistant hosts. The next question that we want to know is whether these uninucleate primary plasmodia in resistant hosts can continue growth and development into zoosporangia and secondary zoospores. In our previous study, most zoosporangial cluster and secondary zoospores in root epidermal cells formation were formed in root hairs or epidermal cells from 4 to 7 dpi in susceptible *Arabidopsis* (Liu et al., 2020). We then investigated and compared the infection process of *P. brassicae* at 7 dpi between resistant and susceptible hosts of *B. napus* or *B. rapa* species by the combination of live cell dyes of HLG, DAPI and FM4-64. In susceptible host plants of *B. napus* Westar, cluster of spherical zoosporangia was observed with abundant lipid droplets and multiple nuclei per zoosporangium in the root epidermal cell (Figure 2B, upper panel). Surprisingly, such structure was also detected in two individual root epidermal cells of resistant plants of *B. napus* ECD10 (Figure 2B, lower panel). In resistant host plants of *B. rapa*, the zoosporangial cluster was also observed from as many as eight individual root epidermal cells (Figure 3A). Importantly, among the zoosporangial cluster, some empty zoosporangia were observed without inclusions and HLG-labeled lipid droplets inside from both resistant hosts and susceptible hosts, suggesting that the secondary zoospores have been released from zoosporangia (Figures 2B, 3A,B). Further examination revealed the presence of free uninucleate secondary zoospores which can be stained by HLG and DAPI and spread around the zoosporangial cluster in the root epidermal cells (Figure 2B, lower panel, and Figures 3A,B). The secondary zoospore was characterized by an ovate head and a visible long flagellum (Figures 2C, 3C). The swimming of secondary zoospores in the lumen of infected root epidermal cells of ECD10 and ECD4 suggested a potential high viability and infectivity (Supplementary Movies S1, S2). These findings demonstrated that *P. brassicae* is able to initiate and complete the primary infection phase to produce free secondary zoospores in resistant hosts. Importantly, the growth and development of *P. brassicae* in resistant hosts does not differ from that in susceptible hosts.

### The Secondary Infection Phase of *P. brassicae* Was Blocked in Both Clubroot-Resistant Species *B. rapa* and *B. napus*

**FIGURE 4:**
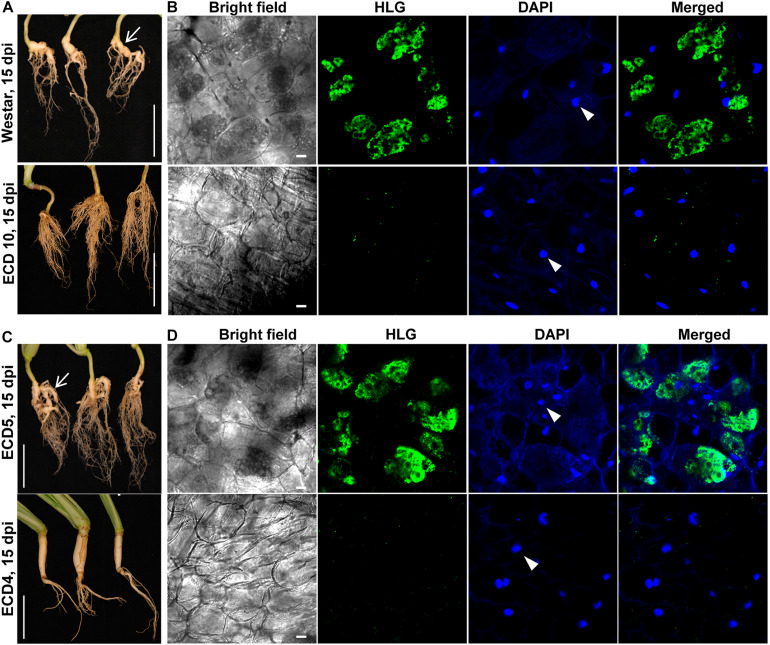
Differences in the secondary infection of *P. brassicae* on susceptible and resistant host plants of *B. napus* and *B. rapa.* The secondary infection of *P. brassicae* was investigated in susceptible host plants of Westar and ECD5 and resistant host plants of ECD10 and ECD4 at 15 dpi. **(A)** Clubroot symptoms appeared on the plant roots of Westar but not ECD10. The plant roots were collected and photographed at 15 dpi. Scale bars: 5 cm. **(B)** FCM images of cortical cells of Westar and ECD10 at 15 dpi. The roots were sectioned by hand and subjected to HLG and DAPI staining and FCM examination. The nuclei of host cortical cells were indicated with white arrows. Scale bars: 10 μm. **(C)** Clubroot symptoms appeared on the plant roots of ECD5 but not ECD4. The plant roots were collected and photographed at 15 dpi. Scale bars: 5 cm. **(D)** FCM images of cortical cells of ECD5 and ECD4 at 15 dpi. The roots were sectioned by hand and subjected to HLG and DAPI staining and FCM examination. The nuclei of host cortical cells were indicated with white arrows. Scale bars: 10 μm.

In the clubroot susceptible host *Arabidopsis*, the secondary infection phase in cortex tissue begun from 7 dpi and resulted in clubroot symptoms at 15 dpi (Liu et al., 2020). Accordingly, we investigated and compared the secondary infection of *P. brassicae* in host species *B. napus* and *B. rapa* at 15 dpi. We found that clubroot symptoms appeared on the roots of two susceptible hosts and were characterized by swollen main roots and small clubs, suggesting the establishment of the secondary infection of *P. brassicae* in cortex tissue (Figures 4A,C, upper panel). The swollen root was sectioned by hand and subjected to HLG and DAPI staining and FCM examination. We found that most cortical cells were colonized by massive secondary plasmodia which enriched in lipid droplets (Figures 4B,D, upper panel). However, we did not observe any club and clubroot symptom on the roots of resistant hosts ECD10 and ECD4 at either15 dpi or 25 dpi (Figures 4A,C, lower panel, and Figure 1). After careful examination of the sections of the main root and all lateral roots from three individual plants of both resistant species, we did not detect any secondary plasmodium in cortical cells (Figures 4B,D). These results suggested that the secondary infection of *P. brassicae* in the cortex tissue is efficiently blocked in those resistant hosts which showed a complete resistance to clubroot disease.

### *P. brassicae* Initiates But Fails to Complete the Primary Infection Phase in Both Clubroot Non-host Crops Wheat and Barley

**FIGURE 5:**
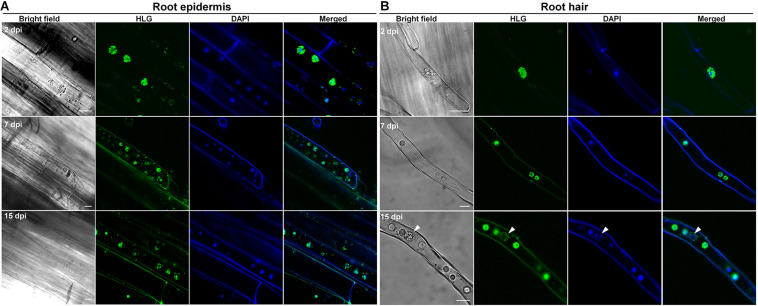
The infection of *P. brassicae* on the plants of non-host crop wheat. Ten-day-old seedlings of wheat were inoculated with resting spores of *P. brassicae*. The roots were collected at 2, 7, and 15 dpi, respectively, and subjected to HLG and DAPI staining and FCM examination. **(A)** FCM images of the parasites in the root epidermal cells at 2, 7, and 15 dpi, respectively. **(B)** FCM images of the parasites in the root hairs at 2, 7, and 15 dpi, respectively. Scale bars: 10 μm.

**FIGURE 6:**
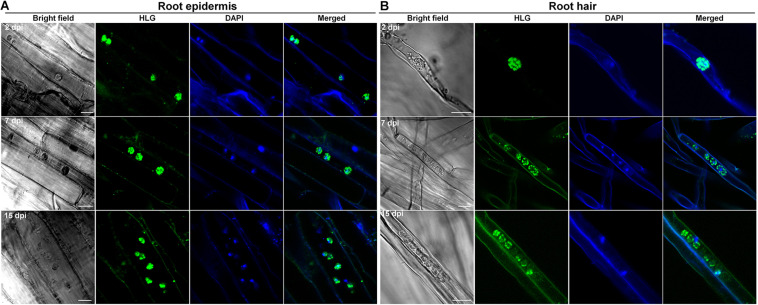
The infection of *P. brassicae* on the plants of non-host crop barley. Ten-day-old seedlings of barley were inoculated with resting spores of *P. brassicae*. The roots were collected at 2, 7 and 15 dpi, respectively, and subjected to HLG and DAPI staining and FCM examination. **(A)** FCM images of the parasites in root epidermal cells at 2, 7, and 15 dpi, respectively. **(B)** FCM images of the parasites in root hairs at 2, 7, and 15 dpi, respectively. Scale bars: 10 μm.

Despite attempts of using clubroot non-host crops like barley, pea, oat, or other non-host species as bait crops to reduce the soil-infested resting spores in several studies, the underlying infection biology of *P. brassicae* on non-hosts is still unclear. Here, we used clubroot non-host crops wheat and barley, the rotation crops of canola in Canada, to investigate the potential infection of *P. brassicae*. The combination of live cell dyes HLG and DAPI was employed to detect *P. brassicae* and we found that at 2 dpi, several HLG-stained uninucleate primary plasmodia of *P. brassicae* were always present in root hairs or epidermal cells of wheat and barley (Figures 5A, 6A, upper panel) and root hairs (Figures 5B, 6B, upper panel), like that were observed in either susceptible or resistant hosts, indicating a successful initiation of the primary infection. Then, we also examined the root hairs and epidermal cells of wheat and barley at 7 dpi using the combination of HLG and DAPI and try to clarify whether zoosporangia and secondary zoospores can be formed. After careful examination of 3 individual roots of wheat or barley, we did not detect any zoosporangial cluster excepting the predominant uninucleate primary plasmodia in root epidermal cells and root hairs (Figures 5B, 6B, middle panel). Similar results were also obtained at 15 dpi (Figures 5B, 6B, lower panel). These results indicated that *P. brassicae* is unable to complete the primary infection phase in non-host crops wheat and barley, despite a successful initiation of the primary infection.

### Quantification of *P. brassicae* Biomass in Susceptible and Resistant Host Plants and Non-host Plants During Its Infection

**FIGURE 7:**
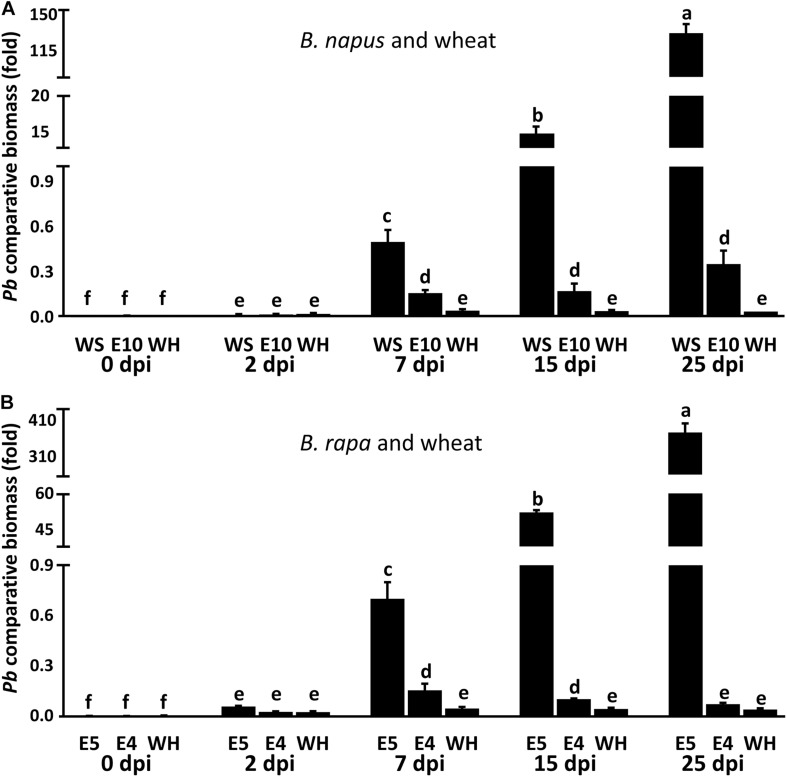
Determination of *P. brassicae* biomass in susceptible and resistant hosts and non-hosts by qPCR. Roots of susceptible hosts Westar and ECD5, resistant hosts ECD10 and ECD4 and non-host crop wheat were collected at 0, 2, 7, 15, and 25 dpi with three repeats, respectively. The total DNA of each root sample was subject to qPCR analysis using specific primers. The primers designed by the sequence of ITS1 and 5.8S rDNA were used for the amplification of *P. brassicae*. The actin genes of *B. napus, B. rapa*, and wheat were used as the internal controls. **(A)** The biomass of *P. brassicae* in susceptible and resistant hosts of *B. napus* and non-host wheat. **(B)** The biomass of *P. brassicae* in susceptible and resistant hosts of *B. rapa* and non-host wheat. The different letters denote statistically significant difference according to one-way ANOVA followed by Tukey’s test (*p* < 0.05). All the primers used here were listed in Supplementary Table S2. WS, Westar; E10, ECD10; WH, wheat; E5, ECD5; E4, ECD4; *Pb*, *P. brassicae*.

As above results revealed, *P. brassicae* can establish the primary infection in both resistant host and non-host plants. However, it is still unclear what level of the biomass of *P. brassicae* accumulates during its infection on resistant host and non-host plants. We then use the quantitative PCR (qPCR) based on DNA to compare and quantify the accumulated biomass of *P. brassicae* parasites between susceptible and resistant hosts or non-hosts. After inoculation, the roots of two resistant hosts ECD10 and ECD4, one non-host crop wheat and two susceptible hosts Westar and ECD5 were harvested at 0, 2, 7, 15, and 25 dpi and used for DNA extraction. The primer pair based on the ITS1 and 5.8S rDNA of *P. brassicae* was used for the determination of accumulated biomass of *P. brassicae* by comparison with the actin gene of plants. We found that the accumulated biomass of *P. brassicae* was almost undetectable in whether susceptible hosts, resistant hosts or non-host plants of wheat at 0 and 2 dpi (Figures 7A,B), indicating that the initiation efficiency of the primary infection of *P. brassicae* has no obvious differences among susceptible and resistant hosts and non-hosts. At the time point of 7 dpi, qPCR revealed that the biomass of *P. brassicae* was significantly increased in susceptible and resistant hosts but not in non-host wheat (Figures 7A,B), supporting that the primary infection phase of *P. brassicae* was completed in resistant hosts but not in non-hosts. From 7 dpi to 25 dpi, qPCR showed that the biomass of *P. brassicae* in susceptible hosts hugely increased, corresponding to the increasing clubroot severity appeared on the roots of susceptible hosts (Figure 7). In contrast, the biomass of *P. brassicae* in resistant hosts and non-hosts was still maintained at a very low level at 15 dpi and 25 dpi and has no significant differences from that at 7 dpi, in favor of that the secondary infection phase failed in those resistant that showed a complete resistance.

## Discussion

Presently, most clubroot-resistant loci or genes are identified from the A genome or the C genome of *Brassica* species (Mehraj et al., 2020). Clubroot resistance from the A genome is always complete and controlled by monogenes, of which *Crr1a* and *CRa* have been isolated (Ueno et al., 2012; Hatakeyama et al., 2013). In contrast, clubroot resistance from the C genome is partial and polygene-controlled. Resistant hosts ECD4 (A genome) and ECD10 (AC genome) showed a complete clubroot resistance. Therefore, in this study, we have investigated the infection biology of *P. brassicae* in those resistant hosts which show a complete rather than a partial clubroot resistance. In susceptible hosts, the life cycle of *P. brassicae* involves in the primary infection phase in the root hair and epidermal cell and the secondary infection in the inner tissue-the cortex which is responsible for the resultant clubroot disease (Tommerup and Ingram, 1971; Ingram and Tommerup, 1972; Kageyama and Asano, 2009; Liu et al., 2020). Different from most plant pathogens, whether eukaryotic or prokaryotic, which are extracellular, *P. brassicae* represents a typical intracellular plant pathogen. Consequently, cellular and molecular mechanisms underlying clubroot resistance may be distinct and partially uncovered in this study.

### A Diagram Summarizing the Life Stages of *P. brassicae* That Are Respectively Blocked in the Resistant Hosts and Non-hosts During Its Life Cycle

**FIGURE 8:**
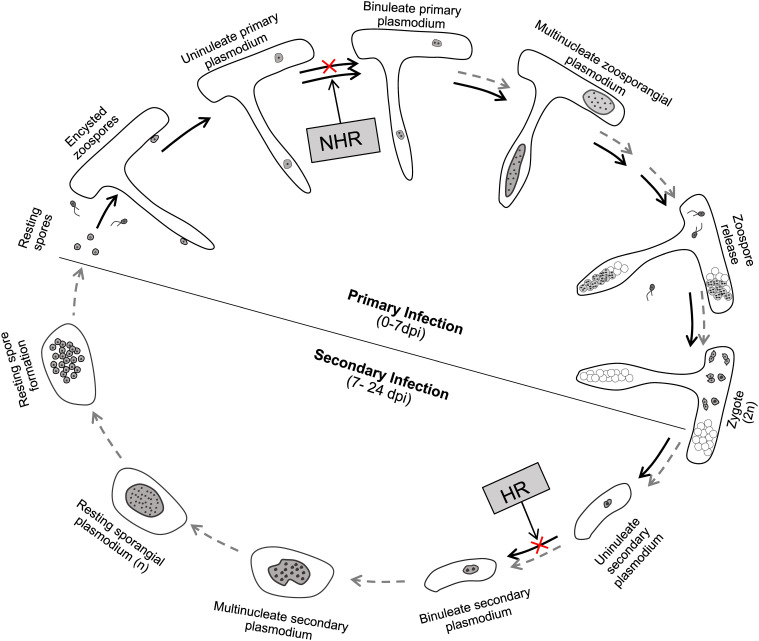
A diagram summarizing the life stages of *P. brassicae* that are blocked in resistant hosts and non-hosts during its infection. The life cycle of *P. brassicae* is based on the model in our previous study with some modifications (Liu et al., 2020). NHR, non-host resistance; HR, host resistance. Black arrows, undergoing development process; gray dashed arrows, failed developmental process.

In a previous study, we have refined the life cycle of *P. brassicae* in susceptible host plants of *Arabidopsis* (Liu et al., 2020). Here, we found that *P. brassicae* performs the same infection progress in susceptible host plants of *B. rapa* and *B. napus*. Therefore, the life cycle of *P. brassicae* proposed in *Arabidopsis* represents a general model in most *Brassicaceae* species, if not all. In this study, we specify the life stages of *P. brassicae* that are blocked in resistant hosts and non-hosts during its life cycle based on our results (Figure 8). *P. brassicae* is able to initiate the primary infection phase in both resistant host and non-host plants, producing uninucleate primary plasmodia in root hairs and epidermal cells. In non-host plants, further growth and development of the uninucleate primary plasmodium are blocked, interrupting the primary infection phase in root hairs or epidermal cells and avoiding the secondary infection in cortex tissue. In resistant host plants, this uninucleate primary plasmodium continues growth and development to yield and release considerable secondary zoospores, completing the primary infection phase. After conjugation of secondary zoospores, the resultant zygote may penetrate the wall as that performed by the primary zoospore and inject the parasite of uninucleate secondary plasmodium into the cortical cell. However, further growth and development of the uninucleate secondary plasmodium may be blocked in the cortical cells of resistant host plants, leading to an interruption of the secondary infection phase.

### Clubroot Resistant Hosts Fail to Restrict the Primary Infection Phase of *P. brassicae* in the Epidermis Tissue

The role of the primary infection phase is to amplify *P. brassicae* from a single primary zoospore to numerous secondary zoospores in the root hair or epidermal cell which facilitates the establishment of the secondary infection in cortex tissue. Our results showed that *P. brassicae* can undergo the primary infection phase well and produced considerable viable secondary zoospores in resistant hosts (Figures 2, 3 and Supplementary Movies S1, S2). It seems that host resistance fails to recognize the intracellular invaded *P. brassicae* parasites and prevent their growth and development in the root hairs and epidermal cells. This is somehow distinct from plant host resistance against extracellular pathogens, which is implemented in the plant epidermis and efficiently prevent the pre-invasion or post-invasion of attempted pathogens (Kourelis and van der Hoorn, 2018). It will be of great interest to understand how *P. brassicae* avoids or suppresses plant surveillance systems and facilitates its primary infection in the resistant hosts in the future.

### Resistant Hosts Contain Infection of *P. brassicae* in the Cortex Tissue

Despite no clear differences in the primary infection of *P. brassicae* between resistant and susceptible varieties, no clubs and massive secondary plasmodia during the secondary infection were observed in the cortical cells of resistant hosts (Figures 4, 5). Therefore, it’s reasonable to infer that the secondary infection of *P. brassicae* was efficiently blocked in the resistant hosts. Unfortunately, we did not observe that which specific life stage in the secondary infection was interrupted. Two assumptions may be responsible in the cortical cells: (1) The failure of initiation of the secondary infection and (2) the inhibition of parasite’s growth and development after the initiation of the secondary infection. Presently, how the secondary zoospore initiates the secondary infection in the cortical cell is unknown. A previous study indicated that the secondary zoospores penetrated the wall of cortical cell and injected the parasites into the cortical cell by a similar machinery that primary zoospores performed to infect the root hair (Aist and Williams, 1971). Considering much less wall width (150–450 nm) between root hairs and cortical cells than the length of primary cyst Stachel structure (700 nm), it could be easy for the secondary cyst, if existing, to penetrate the wall and initiate the secondary infection (Aist and Williams, 1971). Accordingly, it is more acceptable that the inhibition of the growth and development of parasites rather than the failure of initiation of the secondary infection in cortical cells results in interruption of the secondary infection in resistant hosts. This point is also supported by previous studies of which, amoeboid forms of *P. brassicae* were believed to be observed in the cortical cells of resistant host *B. campestris* or *B. oleracea* (Dekhuijzen, 1979; Donald et al., 2008).

In the cortical cells of resistant hosts, how the plant immune surveillance devices recognize the invaded parasites of *P. brassicae* and what defensive responses are triggered are still completely unknown. Although many clubroot resistance loci have been reported in various *Brassica* species, only *Crr1a* and *CRa* are functionally validated by transgene or mutation, and both of them encode proteins carrying a nucleotide binding site (NBS) in the central region and a leucine-rich repeat (LRR) domain at the C-terminus (Ueno et al., 2012; Hatakeyama et al., 2013). *Crr1a* has been demonstrated to be expressed in the stele and cortex of the primary root and hypocotyl where the secondary infection takes place in susceptible hosts. In some cases, dead cortical cells, necrotic tissues, or secondary thickening of xylem were observed in resistant species during the infection of *P. brassicae*, suggesting that hypersensitive responses may be associated with the host resistance against *P. brassicae* (Dekhuijzen, 1979; Fuchs and Sacristán, 1996; Donald et al., 2008). Intriguingly, ROS species were observed to strongly accumulate in the roots of susceptible host *A. thaliana* Col-0 but not in resistant mutant *bik1* (Chen et al., 2016). Understanding of the mechanisms underlying plant resistance against the protist plant pathogen *P. brassicae* will need more works in the future.

### Non-hosts Contain the Infection of *P. brassicae* in Root Epidermis

Several studies have reported that non-host crops could be used as baits to reduce the soil-infested resting spores (Friberg et al., 2006; Ahmed et al., 2011; Hwang et al., 2015). However, the underlying mechanisms are still not clear. In this study, we found that *P. brassicae* is able to initiate the primary infection in both root hairs and epidermal cells of non-host crops wheat and barley, comparable to susceptible hosts (Figures 6, 7). However, unlike resistant hosts, the colonized parasites failed to undergo further growth and development and were stopped by the life stage of uninucleate primary plasmodia. Our results theoretically supported that the rotation of *Brassica* crops with non-host crops contributes to an integrative management to reduce the clubroot disease. In practice, the rotations with non-host crops barley, pea or oat not only reduced clubroot severity and resting spore concentrations, but also increased yield in contrast to continuous cropping of either resistant or susceptible canola (Hwang et al., 2015).

## Data Availability Statement

All datasets generated for this study are included in the article/Supplementary Material.

## Author Contributions

LL was responsible for performing the experiments, conducting the work, and writing this manuscript. LQ was involved in the writing and edition of this manuscript. XC, YZ, and LX were responsible for collecting and analyzing the data. FL, CT, and JH were responsible for analyzing the data and revising the manuscript. SL and YW were responsible for designing, advising, and revising this work. All authors contributed to the article and approved the submitted version.

## Conflict of Interest

The authors declare that the research was conducted in the absence of any commercial or financial relationships that could be construed as a potential conflict of interest.

## Supplementary Material

Supplementary Movie 1 Swimming of secondary zoospores in the lumen of root epidermal cell of resistant host ECD4.

Supplementary Movie 2 Swimming of secondary zoospores in the lumen of two adjacent root epidermal cells of resistant host ECD10.

Supplementary Table 1 Clubroot resistance assay on ECD set.

Supplementary Table 2 Information of primers used in this study.
