# Supplementary material for: Comparing the Infection Biology of Plasmodiophora brassicae in Clubroot Susceptible and Resistant Hosts and Non-hosts
Source: Front Microbiol. 2020 Oct 16;11:507036. doi: 10.3389/fmicb.2020.507036 (PMC7596292; doi:10.3389/fmicb.2020.507036)
Supplement: Supplementary Table 1 — Clubroot resistance assay on ECD set. [file Table_1.DOCX]

| Species | ECD line | Host code | Cultivar group | Disease scale | | | | | Disease  rate | Disease  index | Binary notation | Nomenc-lature |
| --- | --- | --- | --- | --- | --- | --- | --- | --- | --- | --- | --- | --- |
|  |  |  |  | 0 | 1 | 2 | 3 | total |  |  |  |  |
| Ba | 1 | line a | fodder turnip | 11 | 0 | 0 | 0 | 11 | 0.0% | 0 | 0 | 16 |
|  | 2 | line b | fodder turnip | 9 | 0 | 1 | 0 | 10 | 10.0% | 6.7 | 0 |  |
|  | 3 | line c | fodder turnip | 11 | 1 | 0 | 0 | 12 | 8.3% | 2.8 | 0 |  |
|  | 4 | line r | fodder turnip | 13 | 0 | 0 | 0 | 13 | 0.0% | 0 | 0 |  |
|  | 5 | Granaat | cabbage Pe-Tsai | 0 | 0 | 0 | 10 | 10 | 100.0% | 100 | 16 |  |
| Bn | 6 | Dc 101 | fodder rape Nevin | 1 | 0 | 1 | 6 | 8 | 87.5% | 83.3 | 1 | 15 |
|  | 7 | Dc 119 | Giant rape commercial | 0 | 0 | 0 | 6 | 6 | 100.0% | 100 | 2 |  |
|  | 8 | Dc 128 | Giant Rape selection | 0 | 0 | 0 | 7 | 7 | 100.0% | 100 | 4 |  |
|  | 9 | Dc 129 | New Zealand resistant rape | 0 | 0 | 0 | 6 | 6 | 100.0% | 100 | 8 |  |
|  | 10 | Dc 130 | Swede Wilhelmsburger | 10 | 0 | 0 | 3 | 13 | 23.1% | 23.1 | 0 |  |
| Bo | 11 | Badger Shiper | cabbage | 5 | 3 | 0 | 0 | 8 | 37.5% | 12.5 | 0 | 28 |
|  | 12 | Bindsachsener | cabbage | 6 | 3 | 1 | 0 | 10 | 40.0% | 16.7 | 0 |  |
|  | 13 | Jersey Queen | cabbage | 0 | 0 | 1 | 8 | 9 | 100.0% | 96.3 | 4 |  |
|  | 14 | Septa | cabbage | 4 | 2 | 2 | 2 | 10 | 60.0% | 40 | 8 |  |
|  | 15 | Verheul | fimbriate kale | 0 | 0 | 2 | 4 | 6 | 100.0% | 88.9 | 16 |  |

Note,

Ba, *Brassica rapa*; Bn, *Brassica napus*; Bo, *Brassica oleracea*
