# Supplementary material for: Comparing the Infection Biology of Plasmodiophora brassicae in Clubroot Susceptible and Resistant Hosts and Non-hosts
Source: Front Microbiol. 2020 Oct 16;11:507036. doi: 10.3389/fmicb.2020.507036 (PMC7596292; doi:10.3389/fmicb.2020.507036)
Supplement: Supplementary Table 2 — Information of primers used in this study. [file Table_2.DOCX]

| **Species** | **Accession No.** | **Name** | **Primer** | **Sequence_5'->3'** |  |
| --- | --- | --- | --- | --- | --- |
| *P. brassicae* | AB094984.1 | PbITS1 | PbITS1-QF | GGTATGGTAACAACGGACAAGG | |
|  |  |  | PbITS1-QR | TGTGCCACCCACTGCTATCT |  |
| *B. napus* | BnaA02g00190D | BnActin7 | BnAC7-QF | CTGGAATTGCTGACCGTATGAG |  |
|  |  |  | BnAC7-QR | ATCTGTTGGAAAGTGCTGAGGG |  |
| *T. aestivum* | KC775782.1 | TaActin7 | TaAC7-QF | CGACCGTATGAGCAAGGAGA |  |
|  |  |  | TaAC7-QR | AAAATTCGCCGTTACCTGCTG |  |
